# Clinical effectiveness of chin cup treatment for the management of Class III malocclusion in pre-pubertal patients: a systematic review and meta-analysis

**DOI:** 10.1186/s40510-014-0062-9

**Published:** 2014-12-02

**Authors:** Maria I Chatzoudi, Ioulia Ioannidou-Marathiotou, Moschos A Papadopoulos

**Affiliations:** Komninon 75, Kalamaria 55132 Thessaloniki, Greece; Department of Orthodontics, School of Dentistry, Aristotle University of Thessaloniki, 54124 Thessaloniki, Greece

**Keywords:** Class III malocclusion, Class III treatment, Chin cup, Systematic review, Meta-analysis

## Abstract

**Background:**

Chin cup is regarded as the oldest orthodontic appliance for the management of Class III malocclusion. To assess its clinical effectiveness in pre-pubertal patients, a meta-analysis on specific cephalometric values is attempted.

**Methods:**

Detailed electronic and hand searches with no restrictions were performed up to July 2014. Only randomized controlled trials (RCTs) and cohort studies, i.e. prospective controlled trials (pCCTs) and (retrospective) observational studies (OS), were included. Analyses were performed by calculating the standard difference in means and the corresponding 95% confidence intervals, using the random effects model. Data heterogeneity and risk of bias assessment of the included studies were also performed. Study selection, data extraction and risk of bias assessment were performed twice. The level of significance was set at *P* ≤ 0.05 for all tests, except for heterogeneity (*P* ≤ 0.1).

**Results:**

Seven treated groups from five studies (no RCTs, four pCCTs, one OS) were eligible for inclusion, assessing only the short-term occipital pull chin cup effects. In total, 120 treated patients (mean age: 8.5 to 11 years) compared with 64 untreated individuals (mean age: 7.3 to 9.89 years) were assessed by means of 13 cephalometric variables. The overall quality of these studies was low to medium. In comparison to untreated individuals, the SNB and gonial angles decreased significantly following chin cup use, whereas ANB, Wits appraisal, SN-ML, N-Me and overjet increased. For the rest of the variables, no statistically significant differences were detected.

**Conclusions:**

Although the occipital chin cup affects significantly a number of skeletal and dentoalveolar cephalometric variables, indicating an overall positive effect for the treatment of Class III malocclusion, data heterogeneity and between-studies variance impose precaution in the interpretation of the results.

**Electronic supplementary material:**

The online version of this article (doi:10.1186/s40510-014-0062-9) contains supplementary material, which is available to authorized users.

## Background

A number of appliances are available for the treatment of Class III malocclusion [1–6]. Among them, chin cup holds a premium position as a traditional appliance for the early orthopaedic treatment of Class III malocclusion [3,7,8]. However, a thorough and in-depth investigation of the literature reveals controversies and contradictions regarding both its appropriate use and its clinical effectiveness.

The suggested appropriate age for use varies from 4 [9] to 14 [8] years of age. Patients' sex could also be a factor to consider, since females mature earlier than males. Force magnitude should be small in young patients [3,4] and increase gradually, but the suggested force at the centre of the chin cup ranges from 150 g [9] up to 1,200 g [10]. Suggested hours of wear also range between 8 [9] and 18 h per day [11]. Further, concomitant use of additional appliances like maxillary protraction appliances could significantly affect the results [10,12,13].

Clinical results achieved with the chin cup also constitute a matter of debate. Retardation or even sometimes restriction of mandibular growth is supported by some authors [2,4,5], while such effects are questioned by others [3,8,14,15].

Since no standard protocol has been followed from various clinicians, it is evident that the effectiveness of the chin cup varies according to the exact and individualized way of use and it ranges substantially between investigators from minimal [4] to great [16,17]. Although the approach has been investigated through the years, there is still little evidence concerning its clinical effectiveness under the scope of evidence-based medicine, such as in the form of data synthesis derived from systematic reviews (SRs) or meta-analyses (MAs).

Meta-analyses, being important components of SRs, attempt to combine and summarize both qualitative and quantitative data from multiple studies using sophisticated statistical methodology [18,19]. Such a strategy strengthens evidence, giving the results more statistical power and, therefore, more credibility than the individual studies [18]. According to Victor [20], this kind of approach is recommended when existing literature data is both contradictory and confusing and when clinical benefit could be derived.

The null hypothesis investigated in this study is that the chin cup has no clinical effectiveness on Class III malocclusion.

Therefore, the main (PICO) question this MA aims to answer is as follows: for growing patients presenting Class III malocclusion and/or open bite, could chin cup, as compared with no treatment at all, be beneficial for the improvement of their facial, skeletal and dentoalveolar characteristics in the short and long term?

## Methods

The present MA was undertaken after an *a priori* designed protocol according to the Cochrane Handbook for Systematic Reviews of Interventions version 5.1.0 [21] and presented according to the guidelines of the PRISMA Statement for reporting SRs and MAs of studies evaluating health-care interventions [22].

### Data sources and searches

Systematic searches were conducted for published, unpublished and ongoing studies up to July 2014 to identify potentially relevant studies reporting data from growing patients with Class III malocclusion and/or open bite having received treatment with chin cup appliance (occipital or vertical) for the improvement of their facial, skeletal and dentoalveolar characteristics. Every effort to minimize any possible bias in the location of studies was made, and citations to potentially relevant studies from journal articles, dissertations or conference proceedings were located by searching the corresponding electronic databases.

In addition to the electronic searches, manual searching was also performed for the following journals: *American Journal of Orthodontics and Dentofacial Orthopedics*, *Angle Orthodontist*, *European Journal of Orthodontist*, *Journal of Orofacial Orthopedics* and *Orthopedics and Craniofacial Search*, as well as in the reference list of the full-text articles eligible for inclusion, in an effort to identify and retrieve all possible relevant data. Existing SRs and MAs relevant to this study were identified, and their reference lists were also scanned for additional trials. Conference abstracts were additionally searched and inquired on their current status. Articles published in journals, dissertations and conference proceedings were located from several electronic databases following the use of an appropriately adjusted search strategy for each individual database as shown in Additional file 1: Table S1.

No restrictions were applied concerning publication year, language or status. ‘Grey literature’ (i.e. informally published written material by searching the ‘Digital Dissertations’, ‘Conference Paper Index’ and ‘metaRegister of Controlled Trials’ databases) [23] was also included in our search. If additional information was needed, authors were to be contacted.

### Selection of studies

Studies eligible to be included in this MA were (a) randomized controlled trials (RCTs) and (b) cohort studies, i.e. prospective controlled clinical trials (pCCTs) and (retrospective) observational studies (OS), with matching control samples, investigating the clinical effectiveness of the chin cup used for orthodontic/orthopaedic treatment alone or in combination with removable disocclusion or transversal expansion appliances (such as maxillary bite planes, mandibular bite planes, removable palatal cribs or quad helices) in patients being in the pubertal or pre-pubertal growth spurt (6 to 14 years old) at the start of their treatment. Additional file 2: Table S2 presents in detail the eligibility criteria used in this MA.

Screening of titles, abstracts and full-text citations was performed by two review authors independently (MC and II). Any disagreement was resolved by consulting the third reviewer (MAP). Inter-reviewer agreement on study eligibility was assessed by means of the unweighted Cohen's kappa [24]. Levels of agreement were classified as poor (kappa < 0.00), slight (0.00 < kappa < 0.20), fair (0.21 < kappa < 0.40), moderate (0.41 < kappa < 0.60), substantial (0.61 < kappa < 0.80) and almost perfect (0.81 < kappa < 1.00).

The process of study selection, as well as of data extraction and risk of bias assessment, was not performed blinded, since scientific evidence does not strongly recommend masked assessment [25].

### Data extraction and management

Two reviewers (MC and II) extracted independently study characteristics and outcomes from the included studies in an *a priori* developed extraction form. Any disagreements were resolved after consulting the third reviewer (MAP). The Cohen's kappa statistic was used to assess the level of agreement between the two reviewers.

### Risk of bias (quality evaluation) analysis of the included studies

The risk of bias (quality analysis) for all included studies was performed independently by two reviewers (MC and II), with respect to pre-established characteristics. The risk of bias of RCTs was planned to be assessed with the Cochrane risk of bias tool [21]. The risk of bias of non-randomized studies (pCCTs and OS) was assessed with the Downs and Black checklist [26]. The criteria were grouped in five main domains: reporting, external validity, internal validity - bias, internal validity - confounding, and power. All items were given one point when the respective criterion was fulfilled, except for the ‘power’ domain, in which up to five points could be given, summing up to a maximum of 30 points per article. Serious methodological limitations were judged to exist when a non-randomized study collected less than 17 points on the checklist. Again, any disagreements were resolved by discussion after consulting the third reviewer (MAP), and inter-reviewer agreement for both methods was evaluated by the Cohen's kappa statistic.

### Data synthesis and analysis

Data were summarized and considered suitable for pooling if the corresponding RCTs and cohort studies, i.e. pCCTs or (retrospective) OS, used similar exposures in the same way and reported similar outcomes as provided by lateral cephalometric radiographs. The standard difference in means (SDM) and the corresponding 95% confidence intervals (CIs) were calculated, (a) since possibly different magnification factors of the original lateral cephalometric radiographs might have been used or (b) since cephalometric landmarks used in the primary studies for the common variables examined might have not been defined and measured identically for some cephalometric variables, such as the gonial angle. Soft tissue, cast model and perioral muscular electromyography data analyses were also to be performed, if data were available. The pooled estimate (SDMs) of the examined variables and the corresponding 95% CIs were used to construct a forest plot.

Weighting of the pooled estimates was performed with the random effects model since it was expected that one or more non-RCTs would be included in the analysis [27,28] and because this model takes into account the heterogeneity of the data [27]. In addition, since the observed effect was expected to differ across studies due to sample and implementation differences, the use of this model was regarded as more appropriate [29–31].

If the included studies were less than five, exploratory analyses were to be performed instead. Their derivatives are regarded as secondary in validity and should be viewed with caution [31,32].

Analyses were performed by means of the statistical software ‘Comprehensive Meta-Analysis’ version 2.0 (Biostat Inc., Englewood, NJ, USA). The level of significance was set at *P* ≤ 0.05 except for heterogeneity which was set at *P* ≤ 0.10. *P*-values were two-sided.

### Heterogeneity assessment

To identify the extent of data heterogeneity, the Cochran *Q* test for homogeneity with the corresponding *P*-value was calculated [33–35]. Results were statistically significant where *P* ≥ 0.10 [34,35]. In addition, the *I*^2^ index was applied to measure the within-studies variability, where a value of 0% for the *I*^2^ index equals with no observed heterogeneity, while values of 25%, 50% and 75% equal with low, medium and high heterogeneity, respectively [33]. Due to the random effects model application, *T*^2^ was also used to quantify the impact of heterogeneity between studies [27].

## Results

### Literature flow

**Figure 1:**
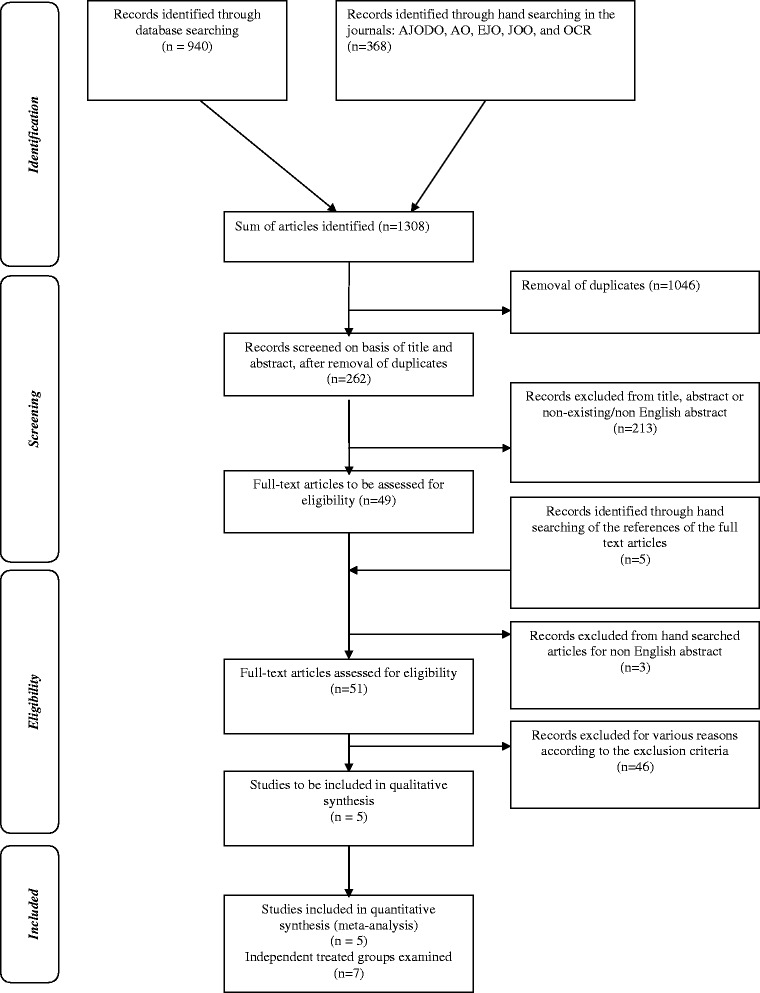
**Flow diagram of the retrieved studies through the selection process.**

Among 1,308 initially identified relevant records (940 through electronic searching and 368 through manual searching of the aforementioned journals), 262 unique citations remained after duplicate removal. From them, 213 were excluded on the basis of the title and abstract, and therefore, 49 articles remained to be assessed on a full text basis. Hand searching on their reference lists revealed five additional relevant records, three from which were excluded due to non-English abstract. Thus, a sum of 51 articles remained for full text eligibility assessment. Application of the detailed inclusion and exclusion criteria resulted in the exclusion of another 46 articles as seen in Additional file 3: Table S3. The numbers of excluded articles, according to the exclusion criteria, are listed in summary in Additional file 4: Table S4. In total, five studies [9–11,13,16] remained for qualitative and quantitative synthesis. All of them were cohort studies and were categorized according to study design into four pCCTs and one OS. Two studies [9,10] examined two independent treated groups versus a control group each, and therefore, the total number of examined independent treated groups versus their corresponding control groups was seven. No RCT was found to be eligible for inclusion in this MA. The flow diagram of the retrieved studies is presented in Figure 1.

Cohen's kappa between the two investigators before reconciliation for data selection was 0.838 (standard error: 0.042) and for data extraction 0.898 (standard error: 0.100), both of which represent an almost perfect agreement.

### Description of studies and baseline characteristics

The characteristics of the included studies [9–11,13,16] are presented in Additional file 5: Table S5.

Although the initial plan was to investigate the short- and long-term effects of both the occipital and the vertical pull chin cup, due to the limited data provided from the included articles, only the short-term occipital pull chin cup effects were finally examined. Consequently, where the term ‘chin cup’ is used thereafter, it is referred to the occipital pull chin cup, and where the term ‘clinical effects’ is used, it is limited to the short-term ones.

Soft tissue, model cast and perioral muscular electromyography data analyses were also not possible to be performed because no such data could be retrieved as appropriate for inclusion and analysis in the present study. Thus, treatment effect comparisons between the experimental groups were considered just for skeletal and dentoalveolar alterations as measured on lateral cephalometric radiographs.

### Risk of bias (quality evaluation) analysis of the included studies

The risk of bias analysis of the included studies according to the Downs and Black [26] checklist is presented in Additional file 6: Table S6.

Cohen's kappa between the two investigators for quality evaluation was 0.750 (standard error: 2.100) for reporting, which is substantial, and 1 (standard error: 0.000) for external validity, internal validity and power, which is perfect. Details can be seen in Additional file 7: Table S7.

### Effectiveness of chin cup treatment

**Figure 2:**
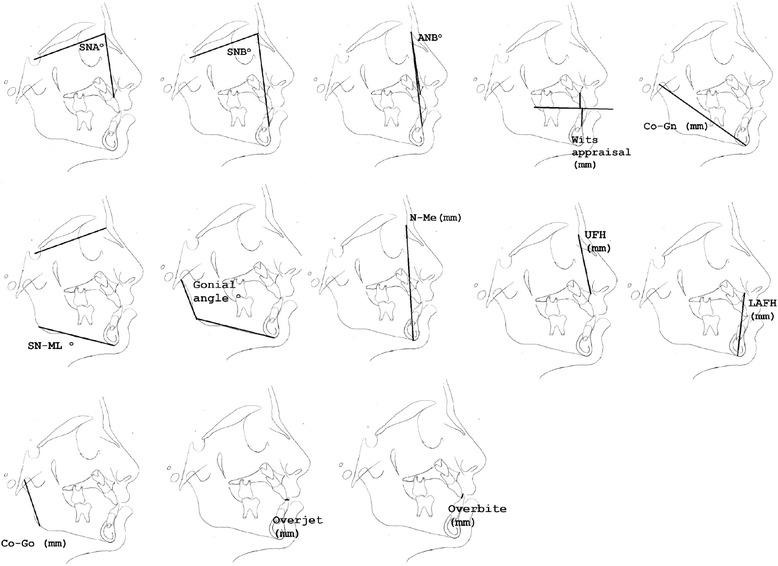
**Cephalometric variables examined in current investigation.**

The common cephalometric variables retrieved from the seven included treated groups and possible to be examined in current MA were the following: (a) *skeletal variables in the sagittal plane*: SNA (°), SNB (°), ANB (°), Wits appraisal (mm) and Co-Gn (mm); (b) *skeletal variables in the vertical plane*: SN-ML (°), gonial angle (°), N-Me (mm), UFH (mm), LAFH (mm) and Co-Go (mm) and (c) *dentoalveolar variables*: overjet (mm) and overbite (mm). These are all presented schematically in Figure 2. The contribution of the original studies to the investigation of each individual cephalometric variable is presented in Table 1.

**Table 1:** **Contribution of the original studies to the investigation of the individual cephalometric variables**

	**Study** ^**a**^							**Sum of studies**	**Sum of separate treated groups**
	**Abdelnaby and Nassar, 2010a [** 10 **]**	**Abdelnaby and Nassar, 2010b [** 10 **]**	**Altuğ et al., 1989** ** [** 16 **]**	**Barrett et al., 2010a** ** [** 9 **]**	**Barrett et al., 2010b** ** [** 9 **]**	**Gökalp and Kurt, 2005** ** [** 11 **]**	**Tuncer et al., 2009** ** [** 13 **]**		
**Force level of the chin cup**	**Heavy**	**Light**	**Not specified**	**Light**	**Light**	**Heavy**	**Light**		
**Additional appliance used**	**Bite plane**	**Bite plane**	**No**	**No**	**Quad helix**	**No**	**Bite plane**		
SNA (°)	Yes	Yes	Yes	Yes	Yes	Yes	Yes	5	7
SNB (°)	Yes	Yes	Yes	Yes	Yes	Yes	Yes	5	7
ANB (°)	Yes	Yes	Yes	Yes	Yes	Yes	Yes	5	7
Wits appraisal (mm)	Yes	Yes	Yes	Yes	Yes	No	No	3	5
Co-Gn (mm)	No	No	No	Yes	Yes	Yes	Yes	3	4
SN-ML (°)	Yes	Yes	Yes	No	No	Yes	Yes	3	5
Gonial angle (°)	Yes	Yes	Yes	Yes	Yes	Yes	No	4	6
N-Me (mm)	Yes	Yes	No	No	No	No	Yes	2	3
UFH (mm)	No	No	No	Yes	Yes	No	Yes	2	3
LAFH (mm)	No	No	No	Yes	Yes	No	Yes	2	3
Co-Go (mm)	No	No	No	Yes	Yes	Yes	No	2	3
Overjet (mm)	No	No	No	Yes	Yes	Yes	No	2	3
Overbite (mm)	No	No	No	Yes	Yes	Yes	No	2	3

^a^Authors in alphabetical order.

**Figure 3:**
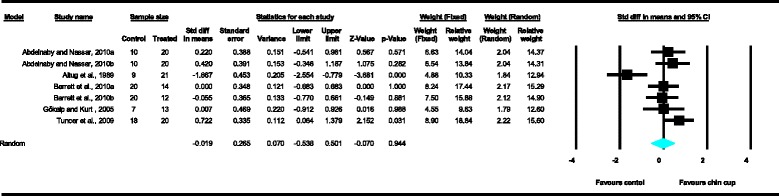
**Forest plot of the cephalometric variable SNA.**

**Figure 4:**
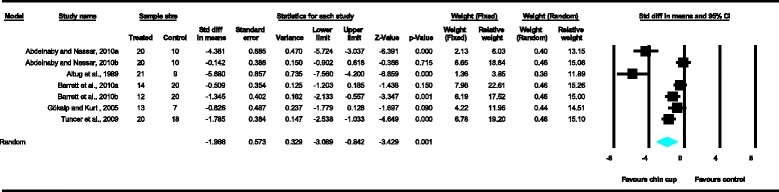
**Forest plot of the cephalometric variable SNB.**

**Figure 5:**
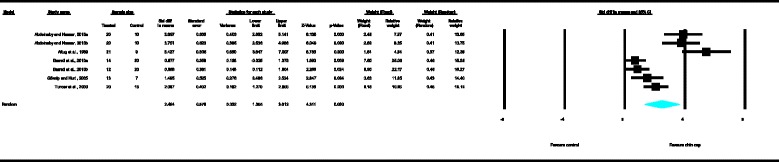
**Forest plot of the cephalometric variable ANB.**

**Figure 6:**
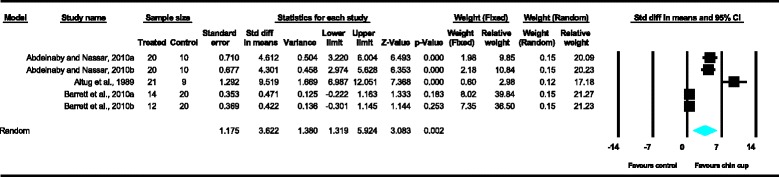
**Forest plot of the cephalometric variable Wits appraisal.**

**Figure 7:**
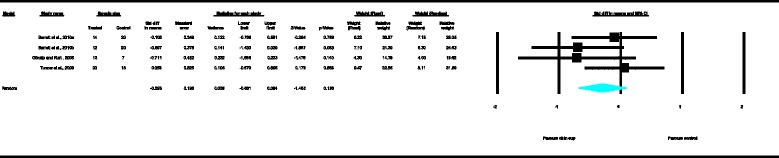
**Forest plot of the cephalometric variable Co-Gn.**

**Figure 8:**
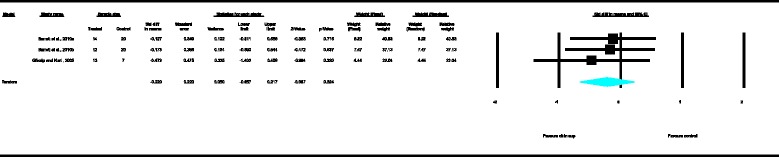
**Forest plot of the cephalometric variable Co-Go.**

**Figure 9:**
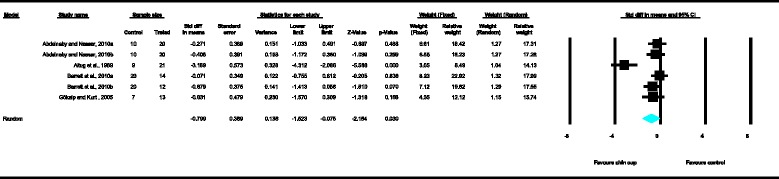
**Forest plot of the cephalometric variable Gonial angle.**

**Figure 10:**
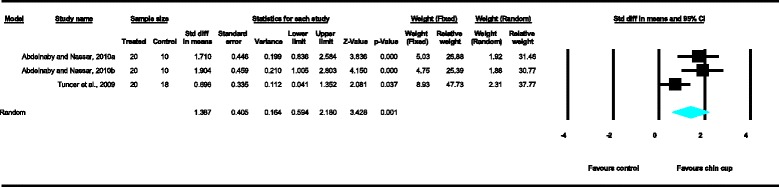
**Forest plot of the cephalometric variable N-Me.**

**Figure 11:**
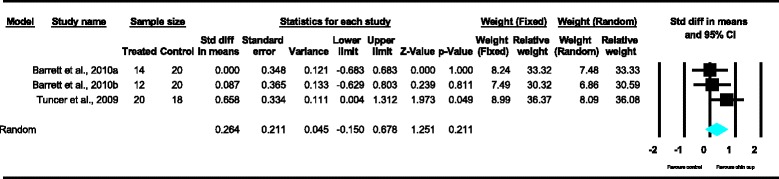
**Forest plot of the cephalometric variable UFH.**

**Figure 12:**
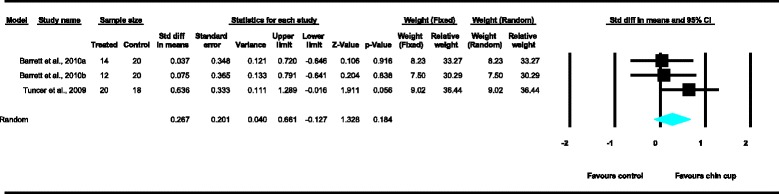
**Forest plot of the cephalometric variable LAFH.**

**Figure 13:**
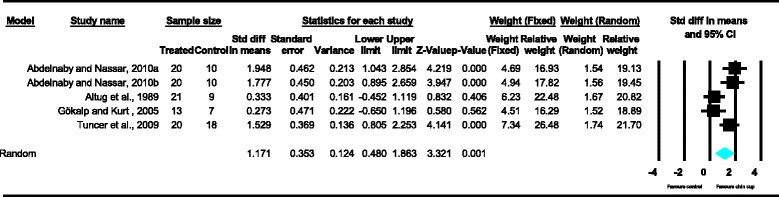
**Forest plot of the cephalometric variable SN-ML.**

**Figure 14:**
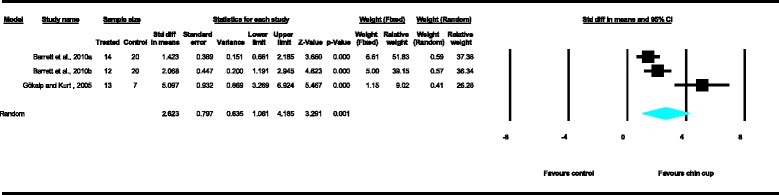
**Forest plot of the cephalometric variable overjet.**

**Figure 15:**
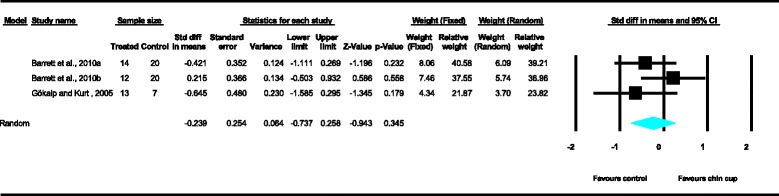
**Forest plot of the cephalometric variable overbite.**

Meta-analyses were performed for the variables SNA, SNB, ANB, Wits appraisal, SN-ML and gonial angle, where data from five or more treated groups derived from the included studies contributed in the analysis. For the rest of the variables, namely Co-Gn, N-Me, UFH, LAFH, Co-Go, overjet and overbite, where data from four or less treated groups contributed in the analysis, exploratory analyses were performed. The summary of pooled estimates of all cephalometric variables under investigation performed with the random effects model is presented in Table 2 and in Figures 3-15. The detailed results of the statistical elaboration of the variables that presented statistically significant differences are presented below.

**Table 2:** **Summary of pooled estimates performed with the random effects model and analysis of heterogeneity**

**Variables**	**Number of treated groups**	**Kind of analysis performed, meta analysis (MA) or exploratory analysis (EA)**	**Heterogeneity**			**Test of null** ***Z*** **(** ***P*** **-value)**	**Standard difference in means**	**95% confidence intervals**	**Significance (** ***P*** **-value)**
			***I*** ^**2**^	***Q*** **(** ***P*** **-value)**	***T*** ^**2**^				
SNA (°)	7	MA	69.362%	19.584 (0.003)	0.338	−0.070 (0.944)	−0.02	−0.54 to 0.50	0.944
SNB (°)	7	MA	90.800%	65.214 (0.000)	2.029	−3.429 (0.001)***	−1.97	−3.09 to −0.84	0.001***
ANB (°)	7	MA	89.614%	57.767 (0.000)	2.029	4.311 (0.000)***	2.48	1.36 to 3.61	0.000***
Wits appraisal (mm)	5	MA	95.645%	91.844 (0.000)	6.364	3.083 (0.002)**	3.62	1.32 to 5.93	0.002**
Co-Gn (mm)	4	EA	11.164%	3.377 (0.337)	0.018	−1.483 (0.138)	−0.29	−0.68 to 0.09	0.138
SN-ML (°)	5	MA	70.569%	13.591 (0.009)	0.437	3.321 (0.001)***	1.17	0.48 to 1.86	0.001***
Gonial angle (°)	6	MA	78.915%	23.714 (0.000)	0.636	−2.164 (0.030)*	−0.80	−1.52 to 0.08	0.030*
N-Me (mm)	3	EA	65.651%	5.823 (0.054)	0.321	3.428 (0.001)***	1.39	0.59 to 2.18	0.001***
UFH (mm)	3	EA	9.200%	2.203 (0.332)	0.012	1.251 (0.211)	0.26	−0.15 to 0.68	0.211
LFAH (mm)	3	EA	0%	1.944 (0.378)	0	1.328 (0.184)	0.27	−0.13 to 0.66	0.184
Co-Go (mm)	3	EA	0%	0.369 (0.832)	0	−0.987 (0.324)	−0.22	−0.66 to 0.22	0.324
Overjet (mm)	3	EA	84.912%	13.256 (0.001)	1.548	3.291 (0.001)***	2.62	1.06 to 4.19	0.001***
Overbite (mm)	3	EA	20.552%	2.517 (0.284)	0.040	−0.943 (0.345)	−0.24	−0.74 to 0.26	0.345

*Statistically significant at *P* ≤ 0.05, **statistically significant at *P* ≤ 0.01, ***statistically significant at *P* ≤ 0.00.

With regard to the skeletal cephalometric changes in the sagittal plane, it was revealed that there was statistically significant reduction in the SNB angle of the patients treated with the chin cup in comparison to the untreated individuals (SDM = −1.97, CI = −3.09 to −0.84, *P* = 0.001), indicating a restriction effect on mandibular growth. This is explicitly shown in Figure 4. In addition, Class III malocclusion of treated patients was significantly improved since there was a statistically significant increase following chin cup use in comparison to untreated individuals to (a) the ANB angle (SDM = 2.48, CI = 1.36 to 3.61, *P* = 0.000) and (b) the Wits appraisal (SDM = 3.62, CI = 1.32 to 5.92, *P* = 0.002) as shown in Figures 5 and 6, respectively. However, for all these three variables, the observed data heterogeneity as well as the between-studies variance was high.

With regard to the skeletal cephalometric changes in the vertical plane, the results of the MA revealed that the SN-ML angle increased significantly as depicted in Figure 13 whereas the gonial angle (Figure 9) decreased significantly in the patients treated with the chin cup as compared with the untreated individuals (SDM = 1.17, CI = 0.48 to 1.86, *P* = 0.001 and SDM = −0.80, CI = −1.52 to −0.08, *P* = 0.030, respectively), indicating a tendency towards an increase of the vertical growth pattern and/or posterior rotation of the mandible. However, data heterogeneity of the included studies was moderate to high, and the between-studies variance was moderate. The tendency towards increase of the anterior face height is further supported by the statistically significant increase of the linear variable N-Me according to the exploratory analysis performed (Figure 10) (SDM = 1.39, CI = 0.59 to 2.18, *P* = 0.001). Moderate data heterogeneity of the included studies and small between-studies variance were also observed here.

As far as the dentoalveolar changes are concerned, the results of the exploratory analysis revealed that there was a statistically significant increase of overjet in the patients treated with the chin cup in comparison to the untreated individuals, clearly depicted in Figure 14 (SDM = 2.62, CI = 1.06 to 4.19, *P* = 0.001), indicating an improvement of the antero-posterior relations of the maxillary and mandibular incisors. Yet, data heterogeneity observed in the included studies, as well as the between-studies variance, was high.

For the rest of the variables, namely SNA, Co-Gn, UFH, LAFH, Co-Go and overbite, no statistically significant differences were derived. This is also presented in the relevant forest plots, depicted in Figures 3, 7, 11, 12, 8 and 15, respectively.

Finally, due to the limited data provided from the included articles, no long-term effects following the use of the occipital chin cup, as well as no short- and long-term effects of the vertical pull chin cup, could be investigated.

## Discussion

According to the results of the current investigation, the null hypothesis was rejected since many of the variables of lateral cephalometric radiographs that were possible to be evaluated presented statistically significant differences.

Patients treated with the chin cup presented, in comparison to untreated individuals, a clockwise rotation of the mandible and an increase in their anterior facial height, both beneficial in solving their skeletal problem.

In detail, statistically significant reduction of the variable SNB indicates a restriction effect on the mandibular growth of growing patients and/or an intense clockwise rotational effect.

The pooled statistically significant increase of the ANB angle entails an improvement of the Class III skeletal relationship of the maxilla and mandible. In the Altuğ et al. [16] study, a much further increase in ANB than in the rest was found. Perhaps this is associated with the individualized protocol applied in that study.

In addition, the Wits appraisal of the treated patients presented a statistically significant increase in comparison to that of the untreated individuals, and this is in accordance with all included studies providing the corresponding treated groups [9,10,16]. Once again, in the study of Altuğ et al. [16], a much greater increase than in the rest was found. Improvement of the Wits appraisal depicts the true and favourable underlying skeletal alterations in the lower face.

The angular variable SN-ML also presented a statistically significant increase in patients treated with chin cup [10,11,13,16]. This is indicative of the increase of the anterior face height that takes place probably due to the backwards and downwards rotation of the mandible [36].

Regarding the statistically significant decrease of the gonial angle, although it is in accordance with the four participating studies providing the corresponding treated groups [9–11,16], in the study of Altuğ et al. [16], a much further reduction was observed than in the others, standing as an outlier. This might indicate that different protocols in the use of the appliance (i.e. different force levels and/or different hours of wear) produce the same result (reduction in this case) but in a very different extent.

Finally, the statistically significant increase of N-Me and overjet, as derived from the corresponding exploratory analyses ([10,13] and [9,11], respectively), further justifies the beneficial use of the chin cup towards the development of a Class I skeletal profile. The observed increase of the anterior facial height could be the result of the clockwise rotation of the mandible whereas the increased overjet might indicate the physiological adaptation of the patients' masticatory system in the new mandibular position where lip and tongue pressures tend towards normal. Yet, although statistical significance was found in these results, exploratory analysis of linear measurements with high heterogeneity imposes precautions in the validity of these results.

A thorough search of the literature revealed that other studies as well, performed under different protocols from the ones implemented in this MA, support many of the aforementioned findings.

The redirection, inhibition and backwards rotation of the mandible and the subsequent decrease of the SNB angle are also supported by the literature [12,36–40]. Increase of the ANB angle supported by many authors in the literature [12,36,37,39,41] may not indicate where exactly the changes were affected [36], but it does mirror the resolution of the skeletal malocclusion that was seen clinically. Sugawara and Mitani [40] however in their study found no significant differences in ANB angle when they compared their Class III subjects with a Class I control group.

Regarding the possible changes in the size of the mandible, the literature supports the opinion that the high density of the bone that forms the mandible seems to react strongly to the chin cup efforts to restrict its growth, permitting only changes to its shape and redirection of its growth [5,12,14,42–46]. Lu et al. [39] after having examined their patients for 5 years found that in the first third of this period, there was a remarkable reduction in the dimension of their mandibles. During the following two thirds of the observation period though, the mandibles increased to their original sizes. On the contrary, Graber [36] found that there was an actual reduction of the size of the mandible.

The closure of the gonial angle due to chin cup use is widely supported by the literature as well [2,12,36,39–43,46] and could be partly explained if the direction of the force applied through the chin cup is considered. The direction of the force passes through the occipital area and the glenoid fossa either via or underneath the condyle and seems to work as a fulcrum, around which the ramus of the mandible tends to rotate [44]. Due to the engagement of the condyle to the articulation and apart from the condyle remodelling to a more forward direction [12], the gonial area also has some freedom to remodelling; thus, it becomes less obtuse [2].

Nevertheless, the N-Me distance seems to increase and many studies in the literature support this finding [2,12,15,39,41,43]. It seems therefore that the simultaneous closure of the gonial angle cannot withstand the increase in anterior face height measured through the variable N-Me. It is possibly the antegonial notch that absorbs much of the applied pressure from the chin cup, thus allowing the deformation of the mandible [40].

### Pros and cons of this MA

The current investigation presents a number of strengths and weaknesses, which are detailed in the following.

Firstly, none of the included studies was a RCT. In addition, from the CCTs included in the analysis, three were appraised as of medium quality [9,10,13] and two as of low [11,16], while the overall estimate was low. The sample sizes were adequate in three of the included studies [10,13,16] and inadequate in the remaining two studies [9,11]. In total, seven treated groups were derived from the five included studies; however, two studies contributed with two independent treated groups each [9,10], whereas the remaining three studies contributed with just one treated group each.

When examining the characteristics of each original study, it was observed that not all treated groups were identical with regard to the amount of force exercised, additional appliances used, duration of treatment, suggested hours of chin cup wear as well as the chronological age of patients. Different protocols in the clinical procedures might have led in different results, however.

For the lateral cephalometric radiographs of the included studies, possibly different magnifications might have been used, since they had been taken at different times in different environments and with different equipment. However, this possible source of bias affected mainly the linear variables, namely Wits appraisal, Co-Gn, N-Me, UFH, LAFH, Co-Go, overjet and overbite, and efforts were made to reduce its impact by the use of the random effects model.

Not all included studies and not all independent treated groups contributed to the statistical elaboration of each of the variables examined. Thus, for the variables Co-Gn, N-Me, UFH, LAFH, Co-Go, overjet and overbite, where four or less treated groups were included, an exploratory analysis was conducted instead of a formal MA. The small number of included treated groups significantly attenuated the validity of the corresponding results and imposed precaution in their interpretation, since they are considered as secondary in comparison with those derived from a formal MA. In addition, high levels of heterogeneity, evident in most of the variables examined, question the validity and reliability of the results.

To balance the reported weaknesses as well as to increase the strength of the current investigation, a strict inclusion-exclusion protocol was applied, according to which specific restrictions to the study designs of the included trials, the participants' characteristics and the principal outcome measures were imposed. Selection of studies, data extraction and management and assessment of the risk of bias of the included studies (quality analysis) were independently performed by two reviewers, while a third one was involved where in doubt to remove subjectivity from the processes. Authors were contacted where no precise information was given. With regard to the statistical computations, the pooled estimate SDM was used instead of the mean difference (MD), to alleviate discrepancies derived from possibly different magnification factors of the lateral cephalometric radiographs evaluated in the original articles, as well as from non-standardized and non-calibrated measurements. Finally, in the current MA, the random effects model was used instead of the fixed effects model in order to take into consideration the data heterogeneity of the included studies, as well as to counterbalance the dominance of the studies with large sample sizes over those with small ones.

## Conclusions

Although the aim of this investigation was to assess the short- and long-term effects of both the occipital and the vertical pull chin cup, due to the lack of appropriate data of the included articles, only the short-term occipital pull chin cup effects were possible to be assessed. In addition, soft tissue, model cast and perioral muscular electromyography data analyses were also not possible to be performed for the same reasons.

Thus, according to the results of this investigation, it can be concluded that following the use of occipital pull chin cup for the short-term management of growing patients with Class III malocclusion before pubertal spurt, an overall significant improvement of the skeletal and dentoalveolar relationships takes place in comparison to untreated individuals. In detail, data elaboration leaded to the following conclusions:

- The skeletal Class III sagittal relationships of the maxilla and mandible are improved.
- The skeletal Class III vertical relationships are also affected towards an increase of the vertical growth pattern, an increase of the anterior face height, and/or posterior rotation of the mandible.
- The antero-posterior relations of the maxillary and mandibular incisors, as indicated by the increase of overjet, are improved.

Nevertheless, the limited number of included studies, the high heterogeneity observed in most of the variables and the linear manner of many of them suggest some precaution in the interpretation of these conclusions. It seems that there is not enough evidence-based data to make definitive recommendations about the chin cup treatment.

More high-quality evidence-based clinical trials with proper design, sample size, appliance use and measurements are needed in the future in order to reach more reliable results concerning the chin cup treatment of Class III malocclusion in the short and the long term.

## Additional files

Additional file 1: Table S1.
**The electronic databases searched, the search strategies used and the corresponding results.** The table presents the strategy followed for the electronic selection of relevant studies at a first level. All the electronic databases searched, the keywords/search strategies used and the results of each database can be seen in this table. This table presents a qualitative evaluation. Additional file 2: Table S2.
**Eligibility criteria used in this meta-analysis.** The table presents the inclusion and exclusion criteria for this meta-analysis according to four separate criteria: outcome, study design, participants' characteristics and principal outcome measures. Additional file 3: Table S3.
**Articles excluded on the basis of the full text from this study and reason for exclusion.** The table presents the full text screened articles that were excluded from the current meta-analysis and the exact reason or study characteristic that leaded to their exclusion. Additional file 4: Table S4.
**Number of the excluded articles according to the exclusion criteria.** This table presents a quantitative evaluation of the excluded articles on full text basis i.e., how many of them were excluded according to the specific criteria. Additional file 5: Table S5.
**Characteristics of the studies included in the meta-analysis.** This table presents the basic characteristics of the included studies permitting their qualitative and quantitative evaluation. More specifically it examines the included studies according to the following sectors: study design, treated sample origin and/or characteristics and sample size, subgroups formed within the study, gender ratio (M / F), and mean age of the treated sample, control sample origin and/or characteristics, control sample size, gender ratio (M / F) and mean age, diagnosis, method of measurement, appliance used, possible additional appliance used, suggested hours of appliance use, treatment follow-ups, treatment duration, control observation and reported effects. Additional file 6: Table S6.
**Risk of bias analysis of the included studies - Downs and Black [** 26 **] scale.** The table presents the assessment of possible bias in the included studies in terms of reporting, external validity, internal validity-bias, internal validity-confounding and power. Additional file 7: Table S7.
**Kappa scores measuring levels of agreement between the two reviewers.** The table presents the levels of agreement between the two reviewers according to kappa score methodology in assessing quality scores of the included articles according to the Downs and Black scale.

## Abbreviations

CCT: controlled clinical trial
CIs: confidence intervals
MA: meta-analysis
MD: mean difference
OS: (retrospective) observational study
pCCT: prospective controlled clinical trial
RCT: randomized clinical trial
SDM: standard difference in means
SE: standard error
SR: systematic review
SNA: angle formed between the cephalometric points S, N and A
SNB: angle formed between the cephalometric points S, N and B
ANB: angle formed between the cephalometric points A, N and B
Wits appraisal: the perpendicular distance of the cephalometric points A and B to the functional occlusal plane
Co-Gn: distance between the cephalometric points Co and Gn
SN-ML: angle formed between the cephalometric planes SN and Go-Me or Go-Gn
Gonial angle: angle formed between the cephalometric points Ar, Go and Me
N-Me: distance between the cephalometric points N and Me
UFH: distance between the cephalometric points N and ANS
LAFH: distance between the cephalometric points ANS and Me
Co-Go: distance between the cephalometric points Co and Go
Overjet: horizontal distance between the cephalometric points “Upper incisor tip” and “Lower incisor tip”
Overbite: vertical distance between the cephalometric points “Upper incisor tip” and “Lower incisor tip”
