# Supplementary material for: Clinical effectiveness of chin cup treatment for the management of Class III malocclusion in pre-pubertal patients: a systematic review and meta-analysis
Source: Prog Orthod. 2014 Dec 2;15(1):62. doi: 10.1186/s40510-014-0062-9 (PMC4250531; doi:10.1186/s40510-014-0062-9)
Supplement: Additional file 1: Table S1. — The electronic databases searched, the search strategies used and the corresponding results. The table presents the strategy followed for the electronic selection of relevant studies at a first level. All the electronic databases searched, the keywords/search strategies used and the results of each database can be seen in this table. This table presents a qualitative evaluation. [file 40510_2014_62_MOESM1_ESM.pdf]

Additional Table 1. The electronic databases searched, the search strategies used and the corresponding results.

Electronic database	Search strategy used	Extend of search	Hits
MEDLINE<br>searched via PubMed<br>(1950 – 10.07.2014)<br>www.ncbi.nlm.nih.gov/pubmed/	"chin cup"[All Fields] OR "chin cups"[All Fields] OR "chin cap"[All Fields] OR "chin caps"[All Fields] OR "chincup"[All Fields] OR "chincups"[All Fields] OR "chincap"[All Fields] OR "chincaps"[All Fields]	In all fields	189
EMBASE<br>searched via ScienceDirect<br>(0.-09.1945 – 10.07.2014)<br>www.embase.com	'chin'/exp AND 'cup' OR ('chin'/exp AND 'cups') OR ('chin'/exp AND 'cap') OR ('chin'/exp AND 'caps') OR 'chincup' OR 'chincups' OR 'chincap' OR 'chincaps' AND [humans]/lim* AND [1-9-1945]/sd NOT [20-9-2011]/sd<br>*limitation=humans	In all fields	80
Cochrane Database of Systematic Reviews<br>searched via The Cochrane Library on 10.07.2014<br>www.thecochranelibrary.com	("chin cup" OR "chin cups" OR "chin cap" OR "chin caps" OR “chincup” OR “chincups” OR “chincap” OR “chincaps”)	In all fields	6
Cochrane Central Register of Controlled Trials<br>searched via The Cochrane Library on 10.07.2014<br>www.thecochranelibrary.com	("chin cup" OR "chin cups" OR "chin cap" OR "chin caps" OR “chincup” OR “chincups” OR “chincap” OR “chincaps”)	In all fields	13
Google Scholar Beta<br>searched on 10.07.2014<br>www.scholar.google.com	“chin cup”<br>“chin cups”<br>“chin cap”<br>“chin caps”<br>“chincup”<br>“chincups”<br>“chincap”<br>“chincaps”	All in title	79<br>2<br>88<br>5<br>41<br>0<br>36<br>0
Web of Science<br>searched on 10.07.2014<br>http://scientific.thomson.com/products/wos/	“chin cup”<br>“chin cups”<br>“chin cap”<br>“chin caps”<br>“chincup”<br>“chincups”<br>“chincap”<br>“chincaps”<br>Filtered by healthcare	In all fields	0<br>0<br>0<br>0<br>0<br>0<br>0<br>0
Evidence-Based Medicine<br>searched on 10.07.2014<br>http://ebm.bmjjournals.com	"chin cup" OR "chin cups" OR "chin cap" OR "chin caps" OR “chincup” OR “chincups” OR “chincap” OR “chincaps”	In all fields	0
Scopus<br>searched on 10.07.2014<br>www.scopus.com	("chin cup" OR "chin cups" OR "chin cap" OR "chin caps" OR “chincup” OR “chincups” OR “chincap” OR “chincaps”)	In all fields	190
LILACS database<br>searched on 10.07.2014<br>http://bases.bvs.br	Abstract=(chin and cup) or (chin and cups) or (chin and cap) or (chin and caps) or (chincup) or (chincups) or (chincap) or (chincaps) AND Group=Humans*<br>*Limitation=humans	In abstract	3

Bibliografia Brasileira de Odontologia<br>searched on 10.07.2014<br>http://pesquisa.bvs.br/brasil/index.php	(chin and cup) or (chin and cups) or (chin and cap) or (chin and caps) or (chincup) or (chincups) or (chincap) or (chincaps)	In abstract	1
Ovid database<br>searched via Heal-link on 10.07.2014<br>http://ovidsp.ovid.com/autologin.html	chin-cup or chin-cups or chin-cap or chin-caps or chincup or chincups or chincap or chincaps	In all fields	0
Bandolier<br>searched on 10.07.2014<br>http://www.medicine.ox.ac.uk/bandolier	("chin cup", "chin cups", "chin cap", "chin caps", “chincup”, “chincups”, “chincap”, “chincaps”)	In all fields	0
Atypon Link<br>searched on 10.07.2014<br>www.atypon-link.com	((chin cup) OR (chin cups) OR (chin cap) OR (chin caps) OR chincup OR chincups OR chincap OR chincaps)	In title, keywords, abstract	0
African Journals Online<br>searched on 10.07.2014<br>www.ajol.info	“chin cup” OR “chin cups” OR “chin cap” OR “chin caps” OR “chincup” OR “chincups” OR “chincap” OR “chincaps”	In all fields	0
Digital Dissertations<br>searched via UMI ProQuest on 10.07.2014<br>http://proquest.umi.com/pqdweb?RQT = 302&cfc = 1	("chin cup" or "chin cups" or "chin cap" or "chin caps" or “chincup” or “chincups” or “chincap” or “chincaps”)	In all fields (Databases: Dissertations &	30
Conference Paper Index<br>searched via Cambridge Scientific Abstracts (1982 –10.07.2014)<br>http://journals.cambridge.org/action/search	(chin cup) OR (chin cups) OR (chin cap) OR (chin caps) OR chincup OR chincups OR chincap OR chincaps	In all fields	0
metaRegister of Controlled Trials (all registers active and archived)<br>searched on 10.07.2014	(chin cup) OR (chin cups) OR (chin cap) OR (chin caps) OR chincup OR chincups OR chincap OR chincaps	In all fields	1
German National Library of Medicine (ZB MED)<br>searched on 10.07.2014<br>http://www.medpilot.de	"chin cup" OR "chin cups" OR "chin cap" OR "chin caps" OR “chincup” OR “chincups” OR “chincap” OR “chincaps”	In all fields	176
**Sum**			**940**
