# Supplementary material for: Clinical effectiveness of chin cup treatment for the management of Class III malocclusion in pre-pubertal patients: a systematic review and meta-analysis
Source: Prog Orthod. 2014 Dec 2;15(1):62. doi: 10.1186/s40510-014-0062-9 (PMC4250531; doi:10.1186/s40510-014-0062-9)
Supplement: Additional file 2: Table S2. — Eligibility criteria used in this meta-analysis. The table presents the inclusion and exclusion criteria for this meta-analysis according to four separate criteria: outcome, study design, participants' characteristics and principal outcome measures. [file 40510_2014_62_MOESM2_ESM.pdf]

Additional Table 2. Eligibility criteria used in this meta-analysis.

Criteria category	*Inclusion criteria*	*Exclusion criteria*
Outcome	Studies investigating the clinical effectiveness of chin cup on growing patients with Class III malocclusion, alone or in combination with removable dissocclusion or transversal expansion appliances (measured with lateral cephalometric analyses or dental cast analyses)	Investigations not relevant to the study
Study design	Randomized controlled trials (RCTs)<br>Cohort studies:<br>i. Prospective controlled clinical trials (pCCTs)<br>ii. (retrospective) Observational Studies (OS)	Unsupported opinions of expert<br>Replies (to author/editor)<br>Editor's choices<br>Books' abstracts<br>Conferences' abstracts<br>Protocol of clinical procedures<br>Technique description<br>Cross-sectional surveys<br>Uncontrolled cohort studies (prospective or retrospective clinical trials)<br>Case-control observational studies<br>Case series without a control<br>Case reports<br>Reviews<br>Systematic reviews<br>Meta-analysis<br>In vitro studies<br>Animal studies/testing<br>Studies on molecular biology, histology, genetics or engineering<br>Studies on cleft lip and palate and craniofacial anomalies<br>Studies on Class I malocclusion<br>Studies on mandibular or maxillary protraction appliances with or without simultaneous use of chin cup<br>Treatment outcomes given after full orthodontic treatment including chin cup and fixed appliances<br>Geometric or morphometric assessment without cephalometric measurements<br>Studies with no English abstract or no abstract at all
Participants' characteristics	Trials referring to human studies investigating patients during/before pubertal growth spurt (6-14 years old) at the start of treatment	Trials referring to human studies investigating patients by the end or after the pubertal growth spurt (15+ years old) at the start of treatment<br>Studies with no matching control sample<br>Clinical trials with inadequate sample size groups, i.e.,<br>- studies of less than 10 participants<br>- studies not reporting the size of the examined sample
Principal outcome measures	Studies providing measurements of skeletal, dental or soft tissue profile changes as recorded by means of lateral cephalometric analyses, dental cast analyses or electromyographic analyses before and after chin cup treatment in the short- and/or long-term	Studies not providing measurements of skeletal, dental or soft tissue profile changes as recorded by means of lateral cephalometric analyses, dental cast analyses or electromyographic analyses before and after chin cup treatment in the short- and/or long-term
