# Supplementary material for: Clinical effectiveness of chin cup treatment for the management of Class III malocclusion in pre-pubertal patients: a systematic review and meta-analysis
Source: Prog Orthod. 2014 Dec 2;15(1):62. doi: 10.1186/s40510-014-0062-9 (PMC4250531; doi:10.1186/s40510-014-0062-9)
Supplement: Additional file 3: Table S3. — Articles excluded on the basis of the full text from this study and reason for exclusion. The table presents the full text screened articles that were excluded from the current meta-analysis and the exact reason or study characteristic that leaded to their exclusion. [file 40510_2014_62_MOESM3_ESM.pdf]

Additional Table 3. Articles excluded on basis of full-text from this study and reason for exclusion.

Nr	*Study**	*Reason for exclusion*
1	Abu Alhaija ES, Richardson A. Long-term effect of the chincap on hard and soft tissues. Eur J Orthod.1999;21:291-8.	Study with maxillary protraction appliance and simultaneous use of chin cup.
2	Alarcón JA, Bastir M, Rosas A, Molero J. Chincup treatment modifies the mandibular shape in children with prognathism. Am J Orthod Dentofacial Orthop. 2011;140:38-43.	Geometric and morphometric assessment without cephalometric values.
3	Allen RA, Connolly IH, Richardson A. Early treatment of Class III incisor relationship using the chincap appliance. Eur J Orthod.1993;15:371-6.	Study with maxillary protraction appliance and simultaneous use of chin cup.
4	Arat ZM, Akçam MO, Gökalp H. Long-term effects of chin-cap therapy on the temporomandibular joints. Eur J Orthod.2003;25:471-5.	Inadequate data: not given cephalometric values post treatment for control and for treated group.
5	Arman A, Toygar TU, Abuhijleh E. Profile changes associated with different orthopedic treatment approaches in Class III malocclusions. Angle Orthod.2004;74:733-40.	Study without contemporaneous and matching in Angle Class control sample.
6	Arun T, Erverdi N. A cephalometric comparison of mandibular headgear and chin-cap appliances in orthodontic and orthopaedic view points. J Marmara Univ Dent Fac.1994;2:392-8.	Case series without a control.
7	Asensi Cros C. Analysis de prognatismos mandibulares tratados ortodoncicamente: Estudio mediante cefalometria. Dissertation, Valencia (Spain) : Univ. de Valencia; 1987.	Cohort study.<br>Uncontrolled (retrospective) observational study.
8	Baccetti T, Franchi L, Schulz SO, McNamara JA Jr.Treatment timing for an orthopedic approach to patients with increased vertical dimension. Am J Orthod Dentofacial Orthop. 2008;133:58-64.	Study on Class I malocclusion.
9	Basdra EK, Stellzig A, Komposch G. Dentofacial changes in patients with Class III malocclusions treated by a combination of activator and chin-cup appliances. Aust Orthod J. 1997;14:225-8.	Study without contemporaneous and matching in Angle Class control sample.
10	Bawazeer OA. Post-treatment stability of class III treatment using RPE and heavy force chin cup protraction. Dissertation, Illinois (PA): Univ of Illinois at Chicago; 2005.	Cohort study.<br>Uncontrolled (retrospective) observational study.
11	Burns NR. Class III camouflage treatment: A retrospective study. Dissertation, West Virginia (PA): Univ. of West Virginia; 2008.	Study without contemporaneous and matching in Angle Class control sample.
12	De Alba A, Chaconas J, Caputo A, Emison W. Stress distribution under high pull extraoral chin cup traction. A photoelastic study. Angle Orthod. 1982;52:69-78.	Technique description.
13	De Alba A, Chaconas J, Caputo A.Orthopedic effect of the extraoral chin cup appliance on the mandible. American Journal of Orthodontics 1976;69:29-41.	Technique description.
14	Deguchi T, Kitsugi A. Stability of changes associated with chin cup treatment. Angle Orthod. 1996;66:139-45.	Results given after full orthodontic treatment including chin cup and fixed appliances.
15	Deguchi T, Kuroda T, Hunt NP, Graber TM. Long-term application of chincup force alters the morphology of the dolichofacial Class III mandible. Am J Orthod Dentofacial Orthop. 1999;116:610-5.	Results given after full orthodontic treatment including chin cup and fixed appliances.
16	Deguchi T, Kuroda T, Minoshima Y, Graber TM. Craniofacial features of patients with Class III abnormalities: growth-related changes and effects of short-term and long-term chincup therapy. Am J Orthod Dentofacial Orthop. 2002;121:84-92.	Results given after full orthodontic treatment including chin cup and fixed appliances.
17	Deguchi T, McNamara JA. Craniofacial adaptations induced by chincup therapy in Class III patients. Am J Orthod Dentofacial Orthop. 1999;115:175-82.	Study with maxillary protraction appliance and simultaneous use of chin cup.
18	Deguchi T, Uematsu S, Kawahara Y, Mimura H. Clinical evaluation of temporomandibular joint disorders (TMD) in patients treated with chin cup. Angle Orthod. 1998;68:91-4.	Cohort study.<br>Uncontrolled (retrospective) observational study.
19	Gavakos K, Witt E. The functional status of orthodontically treated prognathic patients. Eur J Orthod. 1991;13:124-8.	Cohort study.<br>Uncontrolled (retrospective) observational study.
20	Gökalp H, Arat M, Erden I. The changes in temporomandibular joint disc position and configuration in early orthognathic treatment: a magnetic resonance imaging evaluation. Eur J Orthod. 2000;22:217-24.	Study without matching in Angle Class control sample.
21	Graber W. Hyoid changes following orthopedic treatment of mandibular prognathism. Angle Orthod. 1978;48:33-8.	Cohort study.<br>Uncontrolled (retrospective) observational study.

22	Graber W. Chin cup therapy for mandibular prognathism. Am J Orthod. 1977;72:23-41.	Cohort study. Uncontrolled (retrospective) observational study.
23	Harrison E, Shaw C, Worthington V, Bickley S, Scholey J, O'Brien D. Orthodontic treatment for prominent lower front teeth in children. Protocol, Cochrane Database Syst Rev. 2002, Issue 1.	Protocol of clinical procedures.
24	Heymann GC. Temporary anchorage devices for maxillary protraction: Three-dimensional analysis of treatment outcomes. Dissertation, North Carolina (PA): The Univ. of North Carolina at Chapel Hill; 2008.	Dissertation on maxillary protraction appliance and simultaneous use of chin cup.
25	Lentini-Oliveira D et al. Orthodontic and orthopaedic treatment for anterior open bite in children. Cochrane Database Syst Rev. 2000;18:CD005515.	Systematic review.
26	Lin HC, Chang HP, Chang HF. Treatment effects of occipitomental anchorage appliance of maxillary protraction combined with chincup traction in children with Class III malocclusion. J Formos Med Assoc. 2007;106:380-91.	Study with maxillary protraction appliance and simultaneous use of chin cup.
27	Liu ZP, Li CJ, Hu HK, Chen JW, Li F, Zou SJ. Efficacy of short-term chincup therapy for mandibular growth retardation in Class III malocclusion. Angle Orthod. 2011;81:162-8.	Meta-analysis.
28	Long H, Jian F,Lai W. Weak evidence supports the short-term benefits of orthopaedic treatment for Class III malocclusion in children. Evid Based Dent. 2014;15:21-2.	Meta-analysis.
29	Lu YC, Tanne K, Hirano Y, Sakuda M. Craniofacial morphology of adolescent mandibular prognathism. Angle Orthod.1993;63:277-82.	Study with inadequate data: not given cephalometric values pre and post treatment for control or for treated group, just changes given schematically.
30	Luther F, Layton S, McDonald F. Orthodontics for treating temporomandibular joint (TMJ) disorders. Cochrane Database Syst Rev. 2010, Issue 7.	Systematic review.
31	Matsumoto M, Kanaya M, Itoh T, Kawagoe H, Kamogashira K, Ichikawa K, Himeno R, Hirose T, Kawai S, Yanabu M, et al. Statistical investigation on orthodontic patients who received treatment at Fukuoka Dental College during 15 years. Fukuoka Shika Daigaku Gakkai Zasshi. 1989;16:485-94.	Investigation not relevant to the study.
32	Mimura H, Deguchi T. Morphologic adaptation of temporomandibular joint after chincup therapy. Am J Orthod Dentofacial Orthop. 1996;110:541-6.	Results given after full orthodontic treatment including chin cup and fixed appliances.
33	Murata S, Iwata R, Hayakawa S, Fuwa Y, Goto S, Suzuki N. Long term results of skeletal profile changes occurring from chin cap therapy of Japanese female skeletal Class III cases. Aichi Gakuin Daigaku Shigakkai Shi. 1990;28:573-9.	Cohort study. Uncontrolled (retrospective) observational study.
34	Oda H, Ueda A, Miyagawa Y, Hongou H, Kuroki K, Suzuki M, Suzuki Y. Individual growth study of the effects of chin cap force to the mandible. Nippon Kyosei Shika Gakkai Zasshi. 1989 Jun;48:355-61.	Cohort study. Uncontrolled (retrospective) observational study.
35	Pedrin F, Almeida MR, Almeida RR, Almeida-Pedrin RR, Torres F. A prospective study of the treatment effects of a removable appliance with palatal crib combined with high-pull chincup therapy in anterior open-bite patients. Am J Orthod Dentofacial Orthop. 2006;129:418-23.	Study on Class I malocclusion.
36	Ritucci R, Nanda R. The effect of chin cup therapy on the growth and development of the cranial base and midface. Am J Orthod Dentofacial Orthop. 1986;90:475-83.	Study with inadequate data: not given cephalometric values post treatment for control and for treated group.
37	Sakamoto T, Iwase I, Uka A, Nakamura S. A roentgenocephalometric study of skeletal changes during and after chin cup treatment. Am J Orthod. 1984;85:341-50.	Study without matching in age control sample.
38	Sugawara J, Asano T, Endo N, Mitani H. Long-term effects of chincap therapy on skeletal profile in mandibular prognathism. Am J Orthod Dentofacial Orthop. 1990;98:127-33.	Cohort study. Uncontrolled prospective clinical trial.
39	Tahmina K, Tanaka E, Tanne K. Craniofacial morphology in orthodontically treated patients of class III malocclusion with stable and unstable treatment outcomes. Am J Orthod Dentofacial Orthop. 2000;117:681-90.	Cohort study. Uncontrolled prospective clinical trial.
40	Thilander B. Chin cup treatment for Angle Class III maloclusion: a longitudinal study. Trans Eur Orthod Soc.1965;41:311-27.	Cohort study. Uncontrolled (retrospective) observational study.
41	Thilander B. Treatment of Angle Class III malocclusion with chin cup. Trans Eur Orthod Soc. 1963;39:384-98.	Cohort study. Uncontrolled (retrospective) observational study.

42	Toffol LD, Pavoni C, Baccetti T, Franchi L, Cozza P. Orthopedic treatment outcomes in Class III malocclusion. A systematic review. Angle Orthod. 2008;78:561-73.	Systematic review.
43	Torres F, Almeida RR, de Almeida MR, Almeida-Pedrin RR, Pedrin F, Henriques JF. Anterior open bite treated with a palatal crib and high-pull chin cup therapy. A prospective randomized study. Eur J Orthod. 2006;28:610-7.	Study on Class I malocclusion.
44	Torres FC, Almeida RR, Almeida-Pedrin RR, Pedrin F, Paranhos LR. Dentoalveolar comparative study between removable and fixed cribs, associated to chincup, in anterior open bite treatment. J Appl Oral Sci. 2012;20:531-7.	Study on Class I malocclusion.
45	Watkinson S, Harrison JE, Furness S, Worthington HV. Orthodontic treatment for prominent lower front teeth (Class III malocclusion) in children. Cochrane Database Syst Rev. 2013;30:9.	Meta-analysis.
46	Wendell PD, Nanda R, Sakamoto T, Nakamura S. The effects of chin cup therapy on the mandible: a longitudinal study. Am J Orthod. 1985;87:265-74.	Cohort study.<br>Observational (retrospective) study with non consecutive selection of patients. Inadequate data: not given cephalometric values post treatment for control and for treated group.

**studies in alphabetical order*
