# Supplementary material for: Clinical effectiveness of chin cup treatment for the management of Class III malocclusion in pre-pubertal patients: a systematic review and meta-analysis
Source: Prog Orthod. 2014 Dec 2;15(1):62. doi: 10.1186/s40510-014-0062-9 (PMC4250531; doi:10.1186/s40510-014-0062-9)
Supplement: Additional file 4: Table S4. — Number of the excluded articles according to the exclusion criteria. This table presents a quantitative evaluation of the excluded articles on full text basis i.e., how many of them were excluded according to the specific criteria. [file 40510_2014_62_MOESM4_ESM.pdf]

Additional Table 4. Number of the excluded articles according to the exclusion criteria.

Exclusion Criteria	*Number of Excluded Articles*
Investigations not relevant to the study	69
Unsupported opinions of expert	3
Reviews	1
Protocol of clinical procedures	4
Technique description	4
Uncontrolled cohort studies (prospective or retrospective clinical trials)	32
Case series without a control	12
Case reports	24
Systematic reviews	4
Meta-analysis	3
In vitro studies	4
Animal studies/testing	3
Studies on molecular biology, histology, genetics or engineering	5
Studies on cleft lip and palate and craniofacial anomalies	3
Studies on Class I malocclusion	4
Studies on mandibular or maxillary protraction appliances with or without simultaneous use of chin cup	22
Treatment outcomes given after full orthodontic treatment including chin cup and fixed appliances	7
Geometric or morphometric assessment without cephalometric measurements	2
Studies with no English abstract or no abstract at all	42
Studies not providing measurements of skeletal, dental or soft tissue profile changes as recorded by means of lateral cephalometric analyses or dental cast analyses before and after chin cup treatment in the short- and/or long-term	5
Studies with no matching control sample	4
Total	***257***
