# Supplementary material for: Clinical effectiveness of chin cup treatment for the management of Class III malocclusion in pre-pubertal patients: a systematic review and meta-analysis
Source: Prog Orthod. 2014 Dec 2;15(1):62. doi: 10.1186/s40510-014-0062-9 (PMC4250531; doi:10.1186/s40510-014-0062-9)
Supplement: Additional file 5: Table S5. — Characteristics of the studies included in the meta-analysis. This table presents the basic characteristics of the included studies permitting their qualitative and quantitative evaluation. More specifically it examines the included studies according to the following sectors: study design, treated sample origin and/or characteristics and sample size, subgroups formed within the study, gender ratio (M / F), and mean age of the treated sample, control sample origin and/or characteristics, control sample size, gender ratio (M / F) and mean age, diagnosis, method of measurement, appliance used, possible additional appliance used, suggested hours of appliance use, treatment follow-ups, treatment duration, control observation and reported effects. [file 40510_2014_62_MOESM5_ESM.pdf]

Additional Table 5. Characteristics of the studies included in the meta-analysis.

Nr	*Study**	*Study design*	*Treated Sample origin and/or characteristics*	*Treated sample size*	*Subgroups*	*Gender (M / F)*	*Mean age*	*Control sample origin and/or characteristics*	*Control sample size*	*Gender (M / F)*	*Mean age*	*Diagnosis*	*Method of measurement*	*Appliance used*	*Additional appliance used*	*Suggested hours of use*	*Treatment follow-ups*	*Treatment duration*	*Control observation*	*Reported effects*
1	***Abdelnaby and Nassar (2010)a***	pCCT	Growing patients with skeletal Class III pattern, protrusive mandible and anterior cross bite. Origin not defined. All patients had not passed the peak of pubertal growth spurt.	40	Heavy force occipital pull chin cup and upper anterior bite plane (N=20)	10/10	9.6	The same origin with the treated group	10	5/5	9.2	Class III	Lateral cephalometric radiographs, hand-wrist films.	Heavy force occipital pull chin cup (600 gr per side).	Acrylic upper bite plane.	14h/day	1 year	Not defined.	1 year	Significant increase in ANB, Wits appraisal, anterior face height and mandibular plane angle. Significant decrease in SNB and ramus height. Significant increase in retroclination of mandibular incisors.
	Abdelnaby and Nassar (2010)b				Light force occipital pull chin cup and upper bite plane (N=20)	9/11	10.1							Light force occipital pull chin cup (300 gr per side).	Acrylic upper bite plane.					Significant increase in ANB, Wits appraisal, anterior face height and mandibular plane angle. Significant decrease in SNB but less decrease in ramus height. Further retroclination of mandibular incisors.
2	***Altuğ et al. (1989)***	pCCT	Patients with skeletal Class III pattern from the Orthodontic Department in Ankara University.	21	Not specified	11/10	11	Patients with skeletal Class III pattern form from the Orthodontic Department in Ankara University	9	4/5	9.7	Class III	Lateral cephalometric radiographs.	Occipital pull chin cup. Force not specified.	-	Not defined.	0.7years	Not defined.	0.7 years	Significant increase in ANB and in Wits appraisal. Significant decrease in SNB and maxillary plane angle. Decrease in mandibular dimensions Pg-ramus inferior tangent point, Pg-ramus superior tangent point and Pg-Cd.
3	***Barrett et al. (2010)a***	pCCT	Consecutively treated patients with Class III pattern and Witts >-2 Origin not defined.	26	Light force chin cup (N=14)	-	8.5	Patients with Class III malocclusion (matched with the treated sample) from the University of Florence and the University of Michigan Growth Study.	20	6/14	7.3	Class III	Lateral cephalometric radiographs.	Light force occipital pull chin cup (75-125 gr per side).	-	At night only.	1 year	2.6 years for boys, 2.4 years for girls.	2.4 years	Significant increase in ANB. Slight increase in Wits appraisal. Slight decrease in SNB. Significant decrease in maxillary plane angle. Significant increase in retroclination of mandibular incisors.
	Barrett et al. (2010)b				Light force chin cup and quadhelix (N=12)	-	8.5							Light force occipital pull chin cup (75-125 gr per side).	Quadhelix					Significant increase in ANB. and in Wits appraisal. Significant decrease in SNB, gonial angle and in mandibular length and maxillary plane angle. Significant increase in protrusion of maxillary incisors. Less increase in retroclination of mandibular incisors.
4	***Gökalp***	pCCT	Patients with	13	No	3/10	9.06	Patients with	7	1/6	8.9	Class	Lateral	Chin cup	-	18	1.7	1.7	1.7	Significant increase in

	and Kurt 2005		mandibular prognathism. Origin not defined.					mandibular prognathism. Origin not defined.				III	cephalometric radiographs, hand-wrist films, unilateral-left sagittal - oblique TMJ magnetic resonance images (MRIs) in a closed mouth position.	(600 gr).		hrs/day	years	years	years	ANB. and in Wits appraisal. Significant decrease in SNB, gonial angle and in mandibular length and maxillary plane angle. Significant increase in protrusion of maxillary incisors. Less increase in retroclination of mandibular incisors.
5	***Tuncer et al. 2009***	OS	Patients with skeletal Class III pattern mandibular prognathism and dental Class III malocclusion, without forward functional displacement, optimal mandibular plane angle and no congenital anomalies. All patients had not passed the peak of pubertal growth spurt. Origin not defined.	20	No	10/10	10.31	Patients with skeletal Class III pattern and dental Class III malocclusion, mandibular prognathism without forward functional displacement, optimal mandibular plane angle and no congenital anomalies. Patients matched with the treated sample according to skeletal developmental stage. Origin not defined.	18	10/8	9.89	Class III	Lateral cephalometric radiographs, hand-wrist films	Light force occipital pull chin cup (300 gr per side).	Lower bite plane.	14-16h/day	0.8 years	0.8 years	0,95 years	Significant increase in ANB, anterior face height, mandibular plane angle, maxillary forward position and in effective maxillary and mandibular length. Significant decrease in SNB and clockwise rotation of the mandible. Increase in pharyngeal dimension and downward movement of the hyoid bone.

Authors are in alphabetical order; M/F, male / female; RCT, Randomized controlled trial; pCCT, Prospective controlled clinical trial; OS, Observational study.
