# Supplementary material for: Clinical effectiveness of chin cup treatment for the management of Class III malocclusion in pre-pubertal patients: a systematic review and meta-analysis
Source: Prog Orthod. 2014 Dec 2;15(1):62. doi: 10.1186/s40510-014-0062-9 (PMC4250531; doi:10.1186/s40510-014-0062-9)
Supplement: Additional file 6: Table S6. — Risk of bias analysis of the included studies - Downs and Black [ 26 ] scale. The table presents the assessment of possible bias in the included studies in terms of reporting, external validity, internal validity-bias, internal validity-confounding and power. [file 40510_2014_62_MOESM6_ESM.pdf]

Additional Table 6. Risk of bias analysis of the included studies - Downs and Black (1998) scale.

<table>
<tr><th colspan="2">Nr.</th><th colspan="2">1</th><th>2</th><th colspan="2">3</th><th>4</th><th>5</th></tr>
<tr><th colspan="2">Study*</th><th colspan="2">Abdelnaby and Nassar 2010</th><th>Altuğ et al. 1989</th><th colspan="2">Barrett et al. 2010</th><th>Gökalp and Kurt 2005</th><th>Tuncer et al. 2009</th></tr>
<tr><td>Reporting</td><td>Is the hypothesis/aim/objective of the study clearly described?</td><td colspan="2">1</td><td>1</td><td colspan="2">1</td><td>1</td><td>1</td></tr>
<tr><td></td><td>Are the main outcomes to be measured clearly described in the Introduction or Methods section?</td><td colspan="2">1</td><td>1</td><td colspan="2">1</td><td>1</td><td>1</td></tr>
<tr><td></td><td>Are the characteristics of the patients included clearly described?</td><td colspan="2">1</td><td>0</td><td colspan="2">0</td><td>0</td><td>1</td></tr>
<tr><td></td><td>Are the functional appliances used clearly described?</td><td colspan="2">1</td><td>0</td><td colspan="2">1</td><td>0</td><td>1</td></tr>
<tr><td></td><td>Are the distributors of principal confounders in each group of subjects to be compared clearly described</td><td colspan="2">1</td><td>0</td><td colspan="2">1</td><td>1</td><td>1</td></tr>
<tr><td></td><td>Are the main findings of the study clearly described?</td><td colspan="2">1</td><td>1</td><td colspan="2">1</td><td>1</td><td>1</td></tr>
<tr><td></td><td>Does the study provide estimates of the random variability in the data for the main outcomes?</td><td colspan="2">1</td><td>1</td><td colspan="2">1</td><td>1</td><td>1</td></tr>
<tr><td></td><td>Have all important adverse events that may be a consequence of functional appliances been reported?</td><td colspan="2">1</td><td>0</td><td colspan="2">0</td><td>1</td><td>1</td></tr>
<tr><td></td><td>Have the characteristics of patients lost to follow-up been described?</td><td colspan="2">0</td><td>0</td><td colspan="2">0</td><td>0</td><td>0</td></tr>
<tr><td></td><td>Have actual probability values been reported for the main outcomes except where the probability value is less than 0.001?</td><td colspan="2">0</td><td>0</td><td colspan="2">1</td><td>1</td><td>1</td></tr>
<tr><td>External validity</td><td>Were the patients asked to participate in the study representative of the entire population from which they were recruited?</td><td colspan="2">0</td><td>1</td><td colspan="2">0</td><td>0</td><td>0</td></tr>
<tr><td></td><td>Were those subjects who were prepared to participate representative of the entire population from which they were recruited?</td><td colspan="2">0</td><td>1</td><td colspan="2">0</td><td>0</td><td>0</td></tr>
<tr><td></td><td>Were the staff, places and facilities where the patients were treated representative of the treatment the majority of patients receive?</td><td colspan="2">0</td><td>1</td><td colspan="2">1</td><td>1</td><td>0</td></tr>
<tr><td>Internal validity - bias</td><td>Was an attempt made to blind study subjects for the intervention they had received?</td><td colspan="2">1</td><td>0</td><td colspan="2">0</td><td>0</td><td>0</td></tr>
<tr><td></td><td>Was an attempt made to blind those measuring the main outcome of the intervention?</td><td colspan="2">0</td><td>0</td><td colspan="2">1</td><td>0</td><td>0</td></tr>
<tr><td></td><td>If any of the results of the study were based on “data dredging”, was that made clear?</td><td colspan="2">1</td><td>1</td><td colspan="2">1</td><td>1</td><td>1</td></tr>
<tr><td></td><td>In trials, do the analyses adjust for different lengths of follow-up of patients, or in case control studies, is the time and period between the intervention and outcome the same for cases and controls?</td><td colspan="2">1</td><td>1</td><td colspan="2">0</td><td>1</td><td>1</td></tr>
<tr><td></td><td>Were the statistical tests used to assess the main outcomes appropriate?</td><td colspan="2">1</td><td>1</td><td colspan="2">1</td><td>1</td><td>1</td></tr>
<tr><td></td><td>Was compliance with the extraoral appliance used reliable?</td><td colspan="2">0</td><td>0</td><td colspan="2">0</td><td>0</td><td>0</td></tr>
<tr><td></td><td>Were the main outcome measures used accurate (valid and reliable)?</td><td colspan="2">1</td><td>1</td><td colspan="2">1</td><td>1</td><td>1</td></tr>
<tr><td>Internal validity – confounding (selection bias)</td><td>Were the patients in different intervention groups recruited from the same population?</td><td colspan="2">1</td><td>1</td><td colspan="2">0</td><td>0</td><td>1</td></tr>
<tr><td></td><td>Were study subjects in different intervention groups recruited over the same period of time?</td><td colspan="2">1</td><td>1</td><td colspan="2">1</td><td>1</td><td>1</td></tr>
<tr><td></td><td>Were study subjects randomized to intervention groups?</td><td colspan="2">1</td><td>0</td><td colspan="2">0</td><td>0</td><td>0</td></tr>
<tr><td></td><td>Was the randomised intervention assignment concealed from both patients and health care staff until recruitment was complete and irrevocable?</td><td colspan="2">0</td><td>0</td><td colspan="2">0</td><td>0</td><td>0</td></tr>
<tr><td></td><td>Was there adequate adjustment for confounding in the analyses from which the main findings were drawn?</td><td colspan="2">1</td><td>0</td><td colspan="2">1</td><td>1</td><td>1</td></tr>
<tr><td></td><td>Were losses of patients to follow-up taken into account?</td><td colspan="2">0</td><td>0</td><td colspan="2">0</td><td>0</td><td>0</td></tr>
<tr><td></td><td></td><td>a</td><td>b</td><td></td><td>a</td><td>b</td><td></td><td></td></tr>
<tr><td>Power</td><td>Did the study (or the independent treated groups of the study where this applicable) have sufficient power to detect a clinically important effect where the probability value for a difference being due to chance is less than 5%?</td><td>4</td><td>4</td><td>4</td><td>2</td><td>1</td><td>2</td><td>4</td></tr>
<tr><td>Sum</td><td></td><td>21</td><td>21</td><td>17</td><td>16</td><td>15</td><td>16</td><td>20</td></tr>
</table>

**Authors in alphabetical order; yes=1, no=0, unable to determine=0.*
Answers are scored 0 or 1, except for one item in the reporting domain, which is scored 0 to 2, and the single item on power, which is scored 0 to 5.
The correspondence between the sample sizes (N) and the power of the study, after applying G-power statistics, ranks as following: N<10 → power=0, 10<N<12 →power=1, 13<N<15 →power=2, 16<N<18 →power=3, 19<N<21 →power=4 and N>21 →power=5.
