# Supplementary material for: Clinical effectiveness of chin cup treatment for the management of Class III malocclusion in pre-pubertal patients: a systematic review and meta-analysis
Source: Prog Orthod. 2014 Dec 2;15(1):62. doi: 10.1186/s40510-014-0062-9 (PMC4250531; doi:10.1186/s40510-014-0062-9)
Supplement: Additional file 7: Table S7. — Kappa scores measuring levels of agreement between the two reviewers. The table presents the levels of agreement between the two reviewers according to kappa score methodology in assessing quality scores of the included articles according to the Downs and Black scale. [file 40510_2014_62_MOESM7_ESM.pdf]

Additional Table 7. Kappa scores measuring levels of agreement between the two reviewers.

		Reporting		***External validity***		***Internal validity - bias***		***Internal validity – confounding (selection bias)***		***Power***	
Nr.	***Study****	*Reviewer #1*	*Reviewer #2*	*Reviewer #1*	*Reviewer #2*	*Reviewer #1*	*Reviewer #2*	*Reviewer #1*	*Reviewer #2*	*Reviewer #1*	*Reviewer #2*
1	Abdelnaby and Nassar 2010	8	8	0	0	5	5	4	4	4	4
2	Altuğ et al. 1989	4	4	2	2	5	5	2	2	4	4
3	Barrett et al. 2010	7	7	0	0	5	5	2	2	1.5	1.5
4	Gökalp and Kurt 2005	6	7	0	0	5	5	2	2	2	2
5	Tuncer et al. 2009	9	9	0	0	4	4	3	3	4	4
Overall estimate		**0.750 (SE=2.100)**		**1 (SE=0.000)**		**1 (SE=0.000)**		**1 (SE=0.000)**		**1 (SE=0.000)***	

**Levels of agreement between in assessing quality scores of the included articles according to the Downs and Black (1998) scale.*
***Authors in alphabetical order.*
SE, Standard error
